# The Insula and Taste Learning

**DOI:** 10.3389/fnmol.2017.00335

**Published:** 2017-11-03

**Authors:** Adonis Yiannakas, Kobi Rosenblum

**Affiliations:** ^1^Sagol Department of Neuroscience, University of Haifa, Haifa, Israel; ^2^Center for Gene Manipulation in the Brain, University of Haifa, Haifa, Israel

**Keywords:** insular cortex, insula, taste, taste circuit, ERK-MAPK, translation regulation, immediate early genes

## Abstract

The sense of taste is a key component of the sensory machinery, enabling the evaluation of both the safety as well as forming associations regarding the nutritional value of ingestible substances. Indicative of the salience of the modality, taste conditioning can be achieved in rodents upon a single pairing of a tastant with a chemical stimulus inducing malaise. This robust associative learning paradigm has been heavily linked with activity within the insular cortex (IC), among other regions, such as the amygdala and medial prefrontal cortex. A number of studies have demonstrated taste memory formation to be dependent on protein synthesis at the IC and to correlate with the induction of signaling cascades involved in synaptic plasticity. Taste learning has been shown to require the differential involvement of dopaminergic GABAergic, glutamatergic, muscarinic neurotransmission across an extended taste learning circuit. The subsequent activation of downstream protein kinases (ERK, CaMKII), transcription factors (CREB, Elk-1) and immediate early genes (c-fos, Arc), has been implicated in the regulation of the different phases of taste learning. This review discusses the relevant neurotransmission, molecular signaling pathways and genetic markers involved in novel and aversive taste learning, with a particular focus on the IC. Imaging and other studies in humans have implicated the IC in the pathophysiology of a number of cognitive disorders. We conclude that the IC participates in circuit-wide computations that modulate the interception and encoding of sensory information, as well as the formation of subjective internal representations that control the expression of motivated behaviors.

## Introduction

Traditionally, sensory cortices have been broadly defined and studied in relation to the encoding of stimuli arising from a single sensory modality. The insular cortex (IC) and the orbitofrontal cortex comprise the primary and secondary taste cortices, respectively, being particularly tuned to receive and integrate inputs relating to the modality (Ogawa et al., 1985; Rolls, 1989; Pritchard et al., 1999; Kobayashi, 2006). Cytoarchitectural studies demonstrate the IC to be evolutionarily conserved among mammals, despite considerable structural modification between orders (Butti and Hof, 2010). The struggle for sustenance, combined with the salience of taste in establishing associations regulating behavior, have likely contributed in the evolution of the region, currently viewed by many as a center facilitating encoding, processing, and integration of sensory information (Dosenbach et al., 2007; Seeley et al., 2007).

The anatomical location and the limited responsiveness of the IC under anesthesia have hindered its initial characterization (Reil, 1809). Intraoperative recordings in the 1950s, suggested the IC to function primarily as a visceral sensory region (Penfield and Faulk, 1955; Saper, 1982). It was not until the 1980s that single unit recordings during taste information processing were demonstrated, first in rats and later in primates (Yamamoto et al., 1984, 1985; Rolls, 1989; Allen et al., 1991). In more recent fMRI studies in humans, responses of the insula were shown to correlate with the subjective intensity of taste delivered (Grabenhorst and Rolls, 2008; Grabenhorst et al., 2008). Neurological conditions associated with defects in taste perception are rare in humans, but have been observed following IC lesions (Pritchard et al., 1999) or seizures (Isnard et al., 2004). Similarly, in rodents, inactivation of the insula has been associated with significant deficits in a number of taste learning paradigms (Schier et al., 2014, 2016; Blonde et al., 2015; Lin et al., 2015).

Composed of three short gyri, and an additional, ventral accessory gyrus, the anterior insula is home to the gustatory cortex (GC), integral to the representation of tastes, oral texture, and temperature (Shura et al., 2014; Rolls, 2015). On the other hand, the posterior insula, composed of two elongated gyri, is primarily involved in autonomic, sensorimotor and nociceptive processing (Craig, 1996; Brooks et al., 2005; Shura et al., 2014). The lobe is further subdivided in the dorso-ventral cytoarchitectural plane, namely granular (gIC), dysgranular (dIC), and agranular layers (aIC) that relate to progressive changes in the laminar organization of the cortex (Penfield and Rasmussen, 1950; Penfield and Faulk, 1955).

In both humans and rodents, taste perception begins in the periphery, with central axons of cranial nerve ganglia relaying information from taste cells within taste buds of the tongue (and oral cavity). Distinct subclasses of taste receptor cells are known to regulate the perception of salty, sweet, sour, bitter and umami taste in rodents (Mueller et al., 2005; Huang et al., 2006; Chandrashekar et al., 2010; Yarmolinsky et al., 2014). Taste information is conveyed to the rostral and lateral nucleus tractus solitaris of the medulla (Carleton et al., 2010). In rodents, unlike primates, where the PBN is by-passed, fibers from the rostral nucleus tractus solitaris (rNTS) project to the parabranchial nucleus (PBN) and subsequently to the ventroposteromedial nucleus of the thalamus (VPMpc). Rodent gustatory fibers in the PBN projecting to the gustatory thalamus terminate in the IC, laterally and adjacent to the olfactory bulb (Carleton et al., 2010). The rodent and primate insula receives main afferents from the basolateral amygdala (BLA), the dorsal thalamus, other sensory cortical regions, but also the lateral hypothalamus (LHA), the ventral tegmental area (VTA), substantia nigra and dorsal raphe nucleus (Mesulam and Mufson, 1982a,b; Allen et al., 1991). Most of these afferents terminate in the posterior gIC, whereas the ventral aIC predominantly receives limbic input.

Chemosensory information, is encoded through time-varying changes in neuronal firing rates of populations of neurons of the GC (Katz et al., 2001, 2002). States of partially coordinated activity last from few to hundreds of milliseconds and suddenly end, transitioning to the next state (Jones et al., 2007). Activation of the insula has been observed within 200 ms of licking a tastant in rodents (Gutiérrez et al., 2010), while taste discrimination occurs within the 400 ms range (Perez et al., 2013). Electrophysiological recordings from GC neurons in rats during intraoral tastant delivery, have further demonstrated this activity to extend up to 2.5 s from licking, incorporating palatability signals relating to the tastant (Sadacca et al., 2012).

*In vivo* electrophysiological recordings in rats have demonstrated neuronal ensembles to respond to all, some or none of the four basic taste qualities (Jones et al., 2007). This view was supported by findings using intrinsic *in vivo* imaging (Accolla et al., 2007), demonstrating spatial segregation of the neural ensembles representing different taste modalities, but no distinct sub-regional segregation. Nonetheless, two tastants of similar hedonic value activated areas in common regions more often than two tastants with opposite hedonic value, likely facilitating discrimination (Accolla et al., 2007). GC spatiotemporal activity patterns have thus been implicated in the encoding of palatability and/or aversiveness of taste (Accolla and Carleton, 2008), even though the precise sources of valence-related information is still debatable. Reciprocal connections of the GC with the amygdalar complex in particular, have been heavily implicated in reward-related encoding (Stone et al., 2011; Jezzini et al., 2013; Haley et al., 2016).

Studies in mice on the other hand, have argued for a spatially hardwired role of insular sub-regions, shaping a gustotopic map of taste qualities (Chen et al., 2011; Peng et al., 2015). Within this gustotopic map, each taste quality is thought to be encoded by distinct cortical fields, whose activation elicits hardwired behavioral responses. Optogenetic activation of brain fields representing sweet or bitter taste at the GC were shown to exert opposing effects in tests of place preference, leading to aversion to the bitter-associated context. Furthermore, stimulated activation of these GC ensembles, overcame orally generated signals of opposing valence, in go/no go drinking tasks (Peng et al., 2015).

This discrepancy could relate to non-specific activation of regions that participate in the encoding of hedonic value or autonomic-visceral output (Accolla et al., 2007; Accolla and Carleton, 2008). Mice lacking receptors for sweet taste (TRP5-/-), still develop a preference for sucrose over water, demonstrating taste preference to be modulated by non-gustatory inputs such as visceral information and caloric content (de Araujo et al., 2008). Indeed, lesion studies in rats have argued aversive taste memories to be most severely disrupted following damage to the posterior GC overlapping with the visceral cortex, than lesions of the GC alone (Schier et al., 2014, 2016).

Earlier *in vivo* imaging studies of intrinsic neuronal activity of the rat GC, demonstrated differential spatial patterns of activation in response to appetitive saccharin and innately aversive quinine (Accolla and Carleton, 2008). However, in animals trained to be averse to saccharin, internal representations exhibited plasticity, resembling quinine responses (Accolla and Carleton, 2008). Further plasticity (to a saccharin-like pattern) was induced by behavioral extinction of saccharin aversion, underlining that GC activity maps are influenced by associated internal states and hedonics (Carleton et al., 2010). In further support, latest studies utilizing anterograde tracers and two-photon imaging, have noted no apparent spatial organization of primary taste qualities in the layers 2/3 of the GC, just posterior to the middle cerebral artery, in male and female mice (Fletcher et al., 2017). Interestingly, neuronal firing at the GC was shown to participate in the encoding of auditory cues predicting tastants, which following learning were sufficient to evoke a gustatory response even when fluid delivery was omitted (Gardner and Fontanini, 2014). Such cue responses are not commonly observed in sensory cortices, and likely underlie the ability of tastants to act as rewards themselves (Samuelsen et al., 2012, 2013).

Thus, despite its prominent role in the sense of taste, in both primates and other mammals, GC activity can be modulated by cross-modal stimuli (Ghazanfar and Schroeder, 2006; Driver and Noesselt, 2009). The multimodality of IC responses, is not necessarily due to the order of taste sensation in relation to other sensory stimuli, as this can be argued for all perceived sensory stimuli (Zhou and Fuster, 2000; de Araujo and Simon, 2009; Vincis and Fontanini, 2016). Out with physiochemical and affective dimensions of taste, single neuron recordings from neurons of the IC encode tactile, thermal and olfactory information from the oral cavity (Yamamoto et al., 1981; Stapleton et al., 2006; Spence, 2015). The rodent and human rostral agranular insula and its connections has also been implicated in sensorimotor and nociceptive processing, as well as pain inhibitory control (Krushel and van der Kooy, 1988; Craig, 2003; Jasmin et al., 2004; Gogolla et al., 2014; Qiu et al., 2014). Studies of the IC in humans in the past 2 decades, have further implicated the region in interoceptive awareness and the formation of subjective internal representations of experiences and feelings guiding cognition and behavior (Damasio, 2003). This proposed role could relate to the ability of the IC to form internal representations of bodily reactions during sensory experiences (Craig, 2002, 2009; Critchley et al., 2005; Kurth, 2010).

Nevertheless, IC lesions do not disrupt feelings, sentience, or the ability to form taste associations, as the cortex is not the only source and processor of information relating to sensory experiences or inner experiences (Philippi et al., 2012; Damasio et al., 2013). Indeed neural substrates for feeling states initially formed subcortically are sufficient for retrieval, despite their long-term storage in the cortex (Cahill and MacGaugh, 1996). The IC could thus be viewed as a hub of integration of information regarding sensory perception, hedonic and novelty aspects of experiences, with bodily information to facilitate decision making and abstract though (Pritchard et al., 1999; Craig et al., 2000; Bushara et al., 2001, 2003; Baliki et al., 2009; Ibanez et al., 2010).

Single unit recordings and analysis of immediate early gene expression, demonstrated cross-modal responses to be present before learning at the GC and to increase in prevalence and specificity following stimulus-taste associations (Saddoris et al., 2009). Integration of sensory and reward stimuli at the IC, is made possible through interactions with the limbic system, the thalamus and the amygdalar complex in particular (Piette et al., 2012; Samuelsen et al., 2013). In humans and rodents, the BLA is understood to respond strongly to pleasant or unpleasant gustatory stimuli specifically, encoding their relative salience (Small et al., 2003; Fontanini et al., 2009). Palatability related inputs at the IC are abolished in response to BLA lesions (Piette et al., 2012), while during novel taste exposure, changes in IC-BLA connectivity shape the valence of the association (Grossman et al., 2008; Stone et al., 2011). The IC-BLA circuit is increasingly implicated in the formation of taste aversions, however, data at this stage remains largely correlative.

A particular gap in the literature appears to be the precise role of GC and its connectivity with other cortical or lower brain regions during the acquisition of familiarity to appetitive tastants (Lin and Reilly, 2012; Lin et al., 2015). Studies aiming to fully examine the causative role of correlative changes in the expression of genes and protein associated with activity and structural plasticity will hopefully shed more light in the neural and molecular mechanisms of novel taste learning (Koh et al., 2003; Doron and Rosenblum, 2010; Bermudez-Rattoni, 2014).

Insights into molecular and neuroanatomical mechanisms through which the insula is able to encode sensory inputs and serve its complex function stem primarily from basic science studies focusing on taste learning (Bermudez-Rattoni and McGaugh, 1991; Rosenblum et al., 1993; Katz and Harris-Warrick, 1999). Understanding the precise neurobiology involved, holds the potential of revealing how the brain is able to encode, consolidate, and evoke salient information, and thereby coordinate behavior. The focus of this review is to describe the molecular and cellular activity evoked in the insula during encoding or retrieval of hedonic, novelty- and internal body state-related taste-learning inputs.

## Taste learning behaviors

Associative learning paradigms such as *classical conditioning* demonstrate the evolutionary crucial ability of animals to link stimulus and outcome, steering behavior toward positive experiences, instinctively or through learning (Darwin, 1859; James, 1884; Pavlov, 1927; Zajonc, 1980). Unlike other associative learning paradigms, taste conditioning can be achieved in rodents upon a single pairing of a tastant (e.g., 0.5% saccharin water) to a moderate dose of a malaise-inducing agent, e.g., LiCl (Nachman, 1970). Consumption of the conditioned stimulus (CS—e.g., saccharin) is associated with the aversive consequences of the unconditioned stimulus (US—e.g., LiCl), resulting in conditioned taste aversion (CTA) acquisition (Bures et al., 1998; Rosenblum, 2008). Rodents, like all mammals, are naturally hesitant to consume a novel taste due to lack of knowledge regarding its post-ingestive consequences, a phenomenon described as taste neophobia (Barnett, 1958; Carroll et al., 1975). Novel stimuli are typically consumed at limited quantities compared to familiar ones, allowing for examination of post-ingestive consequences (Buresova, 1978; Buresova and Bures, 1980). Following familiarization, if tastants are deemed as safe, neophobia is attenuated and consumption increases (Bures et al., 1998). A number of studies have heavily implicated the insula in the encoding and familiarization to novel tastants as well as the acquisition, retention and extinction of aversive taste memories (Bermudez-Rattoni, 2004, 2014; Rosenblum, 2008; Gal-Ben-Ari and Rosenblum, 2012).

Aversion to bitter substances such as quinine and phenylthiocarbamide is unconditionally induced upon encounter through innate hereditary mechanisms in the natural setting (Kutscher and Wright, 1977; Kutscher et al., 1977; Whitney and Harder, 1986). Bitter tastes are naturally avoided, while sweet tastes are preferred (Spector, 2015). Characteristically, quinine hydrochloride, commonly used as a bitter tastant experimentally, represents a measure of taste aversion, generally thought not to be generated through learning (Zhang et al., 2003; Yamamoto, 2006, 2008). Studies in rats indicate that extensive lesions of the GC in rats leave quinine and sucrose responses unaffected, unlike pre-surgically acquired CTA to NaCl (Kiefer and Orr, 1992). Although follow-up studies have indicated that lesions of 91% of the GC also affect behavioral responses to quinine (Bales et al., 2015), it is likely that these results relate to disruption of cells between the taste and visceral area, that receive convergent baroreceptor, chemoreceptor, gustatory and nociceptive inputs (Hashimoto and Spector, 2014). Evidence suggests that regions play a more prominent role in the regulation of innate taste avoidance behavior, such the ventral striatum, the nucleus accumbens (Yamamoto, 2006, 2008; Rotella et al., 2015) and the balance of inputs to the lateral hypothalamus from the parabranchial nucleus and CeA (Riley and King, 2013; Tokita et al., 2014).

CTA acquired in neophobic animals is a more robust conditioned response (CR), in contrast to CTA to familiar tastants (Garcia et al., 1966) where the animal has to modify the existing safe taste memory trace into one associated with malaise (Pavlov, 1927; Lubow, 1973). This behavioral response is defined as latent inhibition of CTA and is thought to involve re-learning rather than erasing of existing memories (De la Casa and Lubow, 1995; Naor and Dudai, 1996). Latent inhibition (LI) has been produced with different classical and instrumental conditioning paradigms (Lubow, 1989). It was first suggested to arise because of reduced salience of the stimulus as a result of past experience in non-reinforced pre-exposures (MacKintosh, 1975). However, LI most closely compares with habituation where a decline in the attentional orienting response, a measure of stimulus associability (Pearce and Hall, 1980; Swan and Pearce, 1988), is observed within and between sessions of stimulus presentation (Hall and Pearce, 1979; Kaye and Pearce, 1987). The associability of a stimulus declines as its associative strength increases (Pearce and Hall, 1980; Swan and Pearce, 1988) while the orienting response to the CS declines as associative strength increases through training (Kaye and Pearce, 1984). Despite their similarities, habituation and LI are differentially affected by changes in intrinsic salience (Hall, 1991) and context change (Hall and Channell, 1985; Rudy and O'Reilly, 1999).

CTA learning is susceptible to extinction (CTAE) over time following repeated unreinforced CS exposures (Buresova and Bures, 1979). Following Extinction, CTA can be reinstated by administration of the US, context change (Rosas and Bouton, 1997) or spontaneously over time, depending on US strength (Rosas and Bouton, 1998). Extinction is a fundamental topic in investigating experimental psychology of learning and learning theory (Pavlov, 1927; Rescorla and Wagner, 1972; Mackintosh, 1983; Bouton, 1994). Experimental extinction does not involve deleting of the original trace, rather relearning in which memories of unreinforced exposures to the CS, dictate expectation and behavior. This view is supported by a number of findings: (a) spontaneous recovery—once extinguished, CRs may partly recover with time without further training (Pavlov, 1927; Brooks et al., 1999), (b) saving—reacquisition of extinguished behavior can be achieved in fewer trials than naive training (Ebbinghaus, 1964), (c) reinstatement—US exposure alone may restore the CR regardless of the time (Rescorla and Heth, 1975) and (d) renewal—switching out of the extinction context causes CR to reemergence (Bouton and Swarzentruber, 1991).

Research in CTAE is expected to contribute to our understanding of brain processes and mechanisms which interact to determine the dominant characters of internal representations modulating behavior. Once acquired emotional associations are not always expressed, yet regulation of emotional expression under varying environmental conditions is essential for mental health (Quirk and Mueller, 2008). Furthermore, this research could contribute to delineating process through which specific aversive, pleasant or emotional memories are consolidated or modified, providing cues as to how individual memory components can be manipulated to treat disease. Even though studies of the mechanisms underlying the extinction and reinstatement of taste aversion memories are still in their early stages, evidence from human patients highlight the process to involve multisensorial encoding and likely the formation of associations with internal states over periods of time (Berlucchi et al., 2004; Brozzoli et al., 2006). As mentioned, studies have implicated molecular signaling cascades and structural plasticity at the IC of rodents (as well as the medial Pre-frontal Cortex (mPFC), Amygdala and Periaqueductal Gray) in the expression of extinction behavior and potential mechanisms of reinstatement (Berman et al., 2003; Mickley et al., 2005, 2011; Accolla and Carleton, 2008; Maroun et al., 2012; Slouzkey et al., 2013). Of particular interest from our point of view, would be how changes in activity or plasticity within distinct components of the taste-learning circuit, for example how the IC-BLA or mPFC-BLA circuits influence the dominance or extinction of specific engrams (Joels and Lamprecht, 2014; Rodriguez-Duran et al., 2017).

## Synaptic plasticity and taste learning

Studies of the role of the insula in taste learning are increasingly revealing of circuit-wide mechanisms through which the brain coordinates continuous processing and updating of relevant sensory information integrating cognition and behavior (Dudai and Eisenberg, 2004; Fontanini et al., 2009; Senn et al., 2014). During memory acquisition, internal representations of novel information are formed at the cellular and molecular level (Muller and Pilzecker, 1900). Information encoded during acquisition remains labile for a period of time allowing for memory consolidation. Consolidation allows the original memory to transform from short- to long-term memory (McGaugh, 2000; Kandel, 2001; Dudai and Eisenberg, 2004). Memory reconsolidation following memory retrieval will once again render the memory engram labile to metaplastic events in relation to the retrieval setting (Bailey et al., 2004). The consolidation of memory can indeed be disrupted by a number of events such as new learning itself, brain cooling, seizures, trauma, and regional lesions (Alberini, 2011). Importantly, studies using treatments disrupting specific cellular processes such as *de novo* RNA or protein synthesis, as well as the expression or function of specific proteins within the insula, have been successful in modulating taste memory consolidation (Rosenblum et al., 1993; Bures et al., 1998; Merhav and Rosenblum, 2006).

Long-term potentiation (LTP) and its reciprocal long-term depression (LTD) represent the most common cellular model for learning and memory (Bliss and Lomo, 1973). Tetanic stimulation of the BLA prior to training promotes LTP in the IC, and enhances CTA memory retention, demonstrating that synaptic plasticity mechanisms could provide the cellular basis for circuit-wide adaptations to learning (Escobar and Bermudez-Rattoni, 2000). In further support, microinfusion of the known modulator of synaptic plasticity (Braham and Messaoudi, 2005), brain-derived neurotrophic factor (BDNF) into the IC, was associated with the induction of LTP in the BLA-IC projection (Escobar et al., 2003), improving CTA consolidation (Castillo et al., 2006). Importantly, IC–LTP and CTA memory exhibit shared molecular mechanisms in the IC, such as NMDAR dependence, activation of ERK1/2, and induction of immediate early genes (IEGs), including *Zif268, Fos, Arc*, and *Homer* (Jones et al., 1999; Rodriguez-Duran and Escobar, 2014).

Another mechanism shared by late phase LTP and long term memory is the requirement for transcription and translation (Davis and Squire, 1984; Pittenger et al., 2000; Costa-Mattioli et al., 2005; Kandel, 2012). In the case of taste memory, regulation of transcription and translation occurs in the IC, altering synaptic efficacy and strength (Jones et al., 1999; Jones and Katz, 2006; Rodriguez-Duran et al., 2011). Signaling pathways that promote plasticity-related changes in protein expression promote the activation of transcription factors, regulating IEGs, such as *Arc* and *c-fos* (Guzowski et al., 1999; Yasoshima et al., 2006a,b; Mickley et al., 2007; Okuno, 2011). The relative expression of IEGs is widely used to discriminate active neurons following learning and memory, and provides an important link between stimulus-dependent changes in neuronal circuit activity and intracellular molecular mechanisms (Melzer and Steiner, 1997).

Phosphorylated CREB promotes the transcription of a number of genes involved in synaptic plasticity including *Arc, Bdnf, c-fos, zif268*, as well as CCAAT/enhancer binding protein (*C/EBP*β) (Sheng et al., 1990; Finkbeiner et al., 1997; Ying et al., 2002; Ma et al., 2011). CREB phosphorylation in the IC is increased following administration of LiCl or CTA, but not in response to novel taste learning (Swank, 2000; Desmedt et al., 2003). Following novel taste learning, an early shared response in the rodent brain includes the phosphorylation of activity and plasticity marker ERK MAPK in the insular cortex and hippocampus, specifically (Yefet et al., 2006). Conversely, CREB and Akt are phosphorylated in the hippocampus and the cortex during aversive taste memory consolidation, but not during novel taste learning (Swank, 2000; Desmedt et al., 2003; Yefet et al., 2006).

Neurotransmission at the IC underlies the appearance of short- and long-term memory traces that correlate with the strength of associative learning, and shape subsequent behavioral responses to the now-familiar tastant (Doron and Rosenblum, 2010; Yamamoto et al., 2010; Adaikkan and Rosenblum, 2015). Nonetheless, taste learning is subserved by an extended integrative circuit. The IC is bidirectionally linked with the parvocellular portion of the ventroposteromedial thalamic nucleus (VPMpc), as well as the Central and Basolateral Amygdala (CeA and BLA), receiving and processing visceral and emotional information (Koh et al., 2003; Bermudez-Rattoni, 2004; Ferreira et al., 2005; Rosenblum, 2008; Lin and Reilly, 2012). More recent studies have suggested myosin II to regulate actin-related rearrangements in synaptic structure at the Infralimbic mPFC during CTAE (Bi et al., 2015). Further studies specific influence of such events on neurotransmission at the IC or indeed the extended taste during the different phases of taste learning would be particularly interesting.

Anatomical and other functional evidence have highlighted the importance of the IC-BLA interactions CTA learning (Miller et al., 1986; Bermudez-Rattoni and McGaugh, 1991; Bielavaska and Roldan, 1996; Reilly and Trifunovic, 2001; Guzman-Ramos and Bermudez-Rattoni, 2012). Lesions of both the IC and BLA (but not the CeA) have been shown to produce stronger impairments in CTA acquisition than when done separately (Gallo et al., 1992; Bielavaska and Roldan, 1996; Miranda and McGaugh, 2004). Furthermore, cellular compartmental analysis of temporal gene transcription by fluorescence *in situ* hybridization (catFISH) in rats, indicated that at the IC and BLA, CS- and US-information encoding is associated with increases in cytoplasmic and nuclear *Arc* mRNA respectively (Barot et al., 2008). Interestingly, convergent encoding of the two stimuli was only observed in sub-populations of neurons of the BLA but not the agranular IC upon CS-US pairing (Barot et al., 2008). As Barot et al. suggested, CS novelty is likely to be instrumental in potentiating both subsequent US neuronal activation, as well as the formation of an association through timely changes in *Arc* mRNA expression and localization at BLA neurons. However, findings from this study also indicated CS-specific increases in cytoplasmic (but not nuclear) *Arc* mRNA at the dysgranular IC, independent of conditioning (Barot et al., 2008). It is thus likely that even though coincidence detection using catFISH in this study was a valid means of CS-US association encoding at the BLA, it did not necessarily encapsulate the spatiotemporal IEG induction pattern employed at the IC during CS-US associations, which can manifest independently of the amygdala (Saddoris et al., 2009). Furthermore, such coincidence detection might indeed take place at a different time point at the IC, as part of the elegantly demonstrated NMDAR-CAMKII-AMPAR-dependent short-term memory trace, required for CS-US association and effective CTA memory formation (Adaikkan and Rosenblum, 2015).

Thus, the two regions do not serve the same function, but are distinct components of the circuit subserving the different phases of taste memory formation and consolidation through plasticity and complex connectivity (Grossman et al., 2008; Kim et al., 2010; Chen et al., 2011). In the subsequent sections we summarize current findings regarding neurotransmitter systems and molecular signaling cascades implicated in the encoding of novel, and aversive taste memories in the IC, and the extended neuronal circuit facilitating their persistence. The final section of the manuscript is devoted to the role of IEG induction at the IC during the novel and aversive taste learning, and how these facilitate memory formation and persistence.

## Novel taste memory encoding and the insula

Taste information is transferred from the oral cavity to the cortex by the main excitatory neurotransmitter of the mammalian brain, glutamate (Rosenblum, 2008; Rondard et al., 2011). Glutamatergic pathways modulate novelty-driven increases in cortical cholinergic activity, mediated by cholinergic projections of the nucleus basalis magnocellularis (NBM) (Rasmusson, 1993; Giovannini et al., 1995; Moor et al., 1998). Lesion-induced damage to the NBM and its projections to the cortex were previously associated with impairments in the performance of operant spatial memory tasks, working memory, novelty encoding, as well as locomotor activity in rats (Dunnet et al., 1991; Dunnet, 1993; Everitt and Robbins, 1997; Sarter and Bruno, 1997; Himmelheber et al., 2001; Pepeu and Giovannini, 2004).

Cholinergic deafferentation of the cortex in combination with the muscarinic antagonist scopolamine in rats disrupted CTA memory acquisition similarly to excitotoxic lesions (Gutiérrez et al., 1999b,c). In another study, *in vivo* microdialysis was utilized to measure extracellular levels of acetylcholine (ACh) in rats, demonstrating that ACh release in the IC increases in response to novel taste presentation and decreases with each subsequent encounter, finally reaching levels observed during water presentation (Miranda and Bermudez-Rattoni, 1999; Miranda et al., 2000). In studies where the NBM afferents to the IC were inactivated prior to taste presentation, ACh release in the IC was inhibited and novel taste learning was impaired (Miranda and Bermudez-Rattoni, 1999; Miranda et al., 2000). Cellular mechanisms downstream of ACh are key in allowing new learning to take place at the cortex. The release and detection of ACh promotes (a) the enhancement of afferent inputs relative to excitatory feedback, (b) persistent spiking required for active maintenance, as well as (c) synaptic modification, (d) while regulating inhibition and θ oscillations (Hasselmo and McGaughy, 2004; Hasselmo and Giocomo, 2006).

ACh produces biphasic changes in the activity of excitatory neocortical neurons, comprised of fast inhibition, followed by slow, potassium channel dependent depolarization of pyramidal cells (McCormick and Prince, 1985, 1986; Delmas and Brown, 2005). Fast inhibition is mediated by both nicotinic (nAChRs) and muscarinic (mAChRs) receptors that increase the excitability and firing rates of dendrite-targeting GABAergic interneurons (Kawaguchi and Kubota, 1997; Ferezou et al., 2002; Couey et al., 2007; Gulledge et al., 2007; Fanselow et al., 2008; Arroyo et al., 2012). Interestingly, the suppressive effect of novelty-specific mAChR activation is limited to excitation of intracortical intrinsic afferents, but does not affect those evoked by thalamocortical afferents, maintaining the cortex in a state of receptiveness (Gil et al., 1997; Kimura et al., 1999; Kimura, 2000).

Inhibitory neurotransmission by GABA, modulates novelty-driven cholinergic activity in the IC through inhibition within cortical interneuron networks in concert with NBM inputs (Wood and Richard, 1982; Zaborsky et al., 1986) or through GABAA receptor activation in the NBM alone (Chu et al., 1990; DeSousa et al., 1994). Electrophysiological studies demonstrated GABAergic modulation and screening of taste information at both the oral cavity as well as the IC (Ogawa et al., 1998). The infusion of GABA or the GABA_A_ receptor agonist muscimol into the NBM results in decreased cortical ACh release (Casamenti et al., 1986), and impairs performance in several paradigms (Majchrzak et al., 1990; Dudchenko and Sarter, 1991; Muir et al., 1992; Pepeu and Giovannini, 2004). This crucial interplay between ACh and GABA neurotransmission in the IC has only recently been comprehensively addressed with regards to taste novelty (Rodriguez-Garcia and Miranda, 2016). Administration of bicuculline, a GABA_A_ receptor antagonist into the IC or the NBM 24 h. prior to novel taste presentation in rats, was of no consequence to the behavioral response or to ACh release. This treatment was nonetheless sufficient to inhibit the physiological suppression of ACh release required for the acquisition of taste familiarity, and inhibited the physiological attenuation of neophobia (AN) (Rodriguez-Garcia and Miranda, 2016).

In addition, there is an interplay between ACh, gamma-aminobutyric acid (GABA), and glutamate in the IC, as administration of the muscarinic agonist carbachol into the IC increases the amplitude of unitary inhibitory post-synaptic currents (uIPSCs) within local interneuron networks, but suppresses uIPSCs in local pyramidal cells (Yamamoto et al., 2010). This ACh-GABA-glutamate interplay in the IC is thought to occur by novelty-driven cholinergic activation that exerts cell-type specific effects on neuronal excitation by manipulating GABA release into interneuron synapses, modulating the excitatory output in the IC (Fujita et al., 2010). Table 1 summarizes the involvement of different neurotransmitter systems in taste learning behaviors.

**Table 1:** Neurotransmission at the IC modulated by Taste Learning.

**Learning paradigm**	**Manipulation**	**Effect**	**References**
Novel Taste Learning	mAChR antagonism	↓	Berman et al., 1998
	mAChR antagonism	↓	Rodriguez-Garcia and Miranda, 2016
	GABA(A) antagonism	–	Rodriguez-Garcia and Miranda, 2016
	GABA(B) antagonism	↓	Wu et al., 2017
	NMDAR NR2B inhibition	↓	Barki-Harrington et al., 2009
	NMDAR antagonism	–	Gutiérrez et al., 2003a
	NMDAR antagonism	–	Rosenberg et al., 2016a
Attenuation of Neophobia	NR2B F1472 mice	↓	David et al., 2014
	Glutamate	↓	Ramirez-Lugo et al., 2015
	mGLUR1 antagonism	↑	Ramirez-Lugo et al., 2015
Latent Inhibition	AMPAR antagonism	↓	Berman et al., 2000
	D1/D5 antagonism	↓	Berman et al., 2000
	GABA(A) antagonism	↓	Berman et al., 2000
	mAChR antagonism	↓	Berman et al., 2000
	mGLUR2/3 antagonism	↓	Berman et al., 2000
	NMDAR antagonism	↓	Berman et al., 2000
	β-Adrenoreceptor antagonism	↓	Berman et al., 2000
CTA Acquisition	AMPAR antagonism	↓	Berman et al., 2000
	D1/D5 antagonism	↓	Berman et al., 2000
	GABA(A) antagonism	↓	Berman et al., 2000
	mAChR antagonism	↓	Berman et al., 2000
	mGLUR2/3 antagonism	↓	Berman et al., 2000
	NMDAR antagonism	↓	Berman et al., 2000
	β-Adrenoreceptor antagonism	↓	Berman et al., 2000
	GABA(B)1b KO mice	↓	Jacobson et al., 2006
	GABA(B)1b KO mice	No CTAE	Jacobson et al., 2006
CTA Consolidation	Glutamate	↑	Guzman-Ramos et al., 2010
	Dopamine	↑	Guzman-Ramos et al., 2012
CTA Retrieval	AMPAR antagonism	↓	Berman et al., 2000
	GABA(A) agonism	↓	Berman et al., 2000

Novelty-driven cholinergic neurotransmission in the cortex is primarily dependent on mAChRs that utilize G proteins as their signaling mechanism (Clarke, 1993). Activation of mAChRs in the IC during the acquisition of novel taste memory acts upstream of protein synthesis and plasticity stimulating pathways (Rosenblum et al., 2000; Rodriguez-Garcia and Miranda, 2016; Rosenberg et al., 2016b). One of these is the mitogen-activated protein kinase (MAPK) pathway, a key regulator of protein synthesis, is a crucial signal transduction cascade in synaptic plasticity and memory consolidation, modulating both early and late phases of learning-associated LTP and LTD (Sweatt, 2001; Rosenblum et al., 2002; Thiels et al., 2002). In addition to translation, ERK/MAPK is one of the kinase signaling cascades that are regulated by second messengers, and in turn regulate a wave of transcriptional activity in response to neuronal stimulation (Thomas and Huganir, 2004; Bluthgen and Legewie, 2008).

Phosphorylation-dependent activation of the extracellular receptor kinase (ERK 1/2) downstream of MAPK/ERK kinase (MEK) is known to alter the translation of mRNA to protein through phosphorylation of the 40S ribosomal protein S6 kinase (RSK) and subsequent activation of ribosomal protein S6 (Pende et al., 2004). In addition, a number of activity-regulated genes have been reported to be dependent upon ERK phosphorylation through direct or indirect interactions with transcription factors such as ELK-1 and CREB (Thomas and Huganir, 2004; Davis and Laroche, 2006).

ERK phosphorylation is required for the physical manifestation of learning at activated synapses, and serves as a hub of plasticity-related neurotransmission (Berman et al., 1998, 2000). mAChR-dependent activation of ERK in the IC during novel taste learning (Berman et al., 1998; Belelovsky et al., 2005) is not accompanied by activation of other related kinases (Rosenblum et al., 2000), and is necessary for IC LTP induction in this context (Jones et al., 1999). ERK activation following taste learning is both time- and region-specific, in that it is observed in the IC 20 min after taste learning, but not necessarily in other memory-related regions (Berman et al., 1998; Swank and Sweatt, 2001; Sweatt, 2001; Yefet et al., 2006). Studies using the muscarinic agonist carbachol have demonstrated this effect to be Src-dependent and partially phosphoinositide-3 kinase (PI3-K)—and Ca^2+^-dependent, but protein kinase C (PKC)-independent (Kim et al., 1999; Rosenblum et al., 2000; Budd et al., 2001). Nonetheless, taste memory formation and stabilization in the IC is subject to perturbation by multiple neurotransmitter systems (Table 2).

**Table 2:** Intracellular Signaling at the IC modulated by the Taste Learning.

**Learning paradigm**	**Signaling molecule**	**Phosphorylation site**	**Effect**	**References**
Novel Taste Learning	eEF2	T56	↑	Belelovsky et al., 2005
	ELK I	S383	↑	Berman et al., 1998
	ERK	T202/Y204	↑	Berman et al., 1998
	ERK	T202/Y205	↑	Swank and Sweatt, 2001
	JNK I/II	T183/Y185	↑	Berman et al., 1998
	MEK	S217/221	↑	Swank and Sweatt, 2001
	mTOR	Ser2448	↑	Belelovsky et al., 2009
	NMDAR NR2B	Y1472	↑	Nunez-Jaramillo et al., 2008
	NMDAR NR2B	Y1473	↑	Barki-Harrington et al., 2009
	p90RSK	S381	↑	Swank and Sweatt, 2001
	PSD-95		↑	Elkobi et al., 2008
	Raf	S259	↑	Swank and Sweatt, 2001
	S6K1	T389	↑	Belelovsky et al., 2005
CTA Acquisition	CREB	S133	↑	Swank, 2000
	NMDAR NR2B	Y1472	↑	Rosenblum et al., 1995
	PKC		↑	Rodriguez-Duran and Escobar, 2014
	PSD-95		↑	Miyakawa et al., 1997
	TrkB	Y	↑	Ma et al., 2011
CTA Consolidation	CaMKIIα	T286	↑	Adaikkan and Rosenblum, 2015

The balance of GABAergic activation within the IC regulates the ability of organisms to attribute salience and encode information regarding sensory stimuli during memory acquisition and retrieval. Microinfusion of the GABAA receptor agonist, muscimol into the IC, suppresses the ability of a novel tastant to increase ERK phosphorylation, inhibiting synaptic plasticity required for taste memory formation (Berman et al., 2000). Conversely, microinfusion of the GABAAR antagonist bicuculline in the IC prior to water drinking, enhanced ERK phosphorylation, but neither affected drinking behavior itself (Berman et al., 2000). More recent studies have further demonstrated muscimol microinfusion into the rat agranular IC to suppress palatability-driven feeding, but not drinking itself (Baldo et al., 2016). This effect was characterized by a decrease in chocolate-shake intake and the duration of time spent ingesting chocolate shake, but did not affect water drinking or ingestion of standard chow in food-deprived rats (Baldo et al., 2016). It is thus likely that cholinergic activation of ERK within the IC during taste memory acquisition not only encompasses encoding of the novelty of the stimulus, but also the qualitative characteristic of the stimulus, which determines its relative consumption in future encounters (Adaikkan and Rosenblum, 2012).

GABAergic IC neurotransmission is also likely to influence the interception of CS-related cues that allow organisms to attribute salience and facilitate rapid decision making and behavioral modifications during sensory experience-based learning and retrieval (Baldo et al., 2016; Rodriguez-Garcia and Miranda, 2016). Muscimol microinfusion into the IC prior to CTA retrieval in rats suppressed aversion to the CS (Berman et al., 2000), but did not induce preference reminiscent of AN reported elsewhere (Lin and Reilly, 2012; Moraga-Amaro et al., 2014; Rodriguez-Garcia and Miranda, 2016). Taken together findings are consistent with GABAergic local circuits generating familiarity signals that inhibit the attribution of salience and palatability-driven behavior. Interestingly, increased GABAergic neurotransmission in the IC has been suggested to contribute to disruption of interoceptive awareness observed in major depression and anorexia nervosa patients (Wiebking et al., 2014).

Inhibitory GABAergic interneurons of the IC have also been involved in off-line processing following novel taste learning (Doron and Rosenblum, 2010). For example, GABAergic, GAD67^+^ neurons residing in the deep layers of the IC were shown to express increased *c-fos* immunoreactivity 2 h following appetitive novel taste learning (Doron and Rosenblum, 2010). GABAergic inhibition has also been observed to increase in response to novel sensory information during off-line processing in other regions responsible for the encoding of other modalities (McCasland and Hibbard, 1997; Staiger et al., 2002; Murayama et al., 2009). Increased IC GABAergic activation following novel taste consumption could be indicative of offline malaise-related computations that are required for AN, as taste information is evaluated in relation to associated lower brain inputs (Dudai and Eisenberg, 2004; Doron and Rosenblum, 2010; Adaikkan and Rosenblum, 2015). One possibility might be that increased IC inhibition subserves the unique attribute of taste to be associated with visceral information in the subsequent hours (Rosenblum, 2008). Interestingly, microinfusion of both GABA_B_R agonists and antagonists into the IC was recently shown to disrupt recognition memory in mice, suggesting GABA_B_R balance in the IC to be critical for memory formation (Wu et al., 2017). GABA_B_R-dependent neurotransmission has been previously reported to promote synaptic plasticity in hippocampal neurons through ERK-dependent mechanisms (Im and Rhim, 2012). However, the relevant contribution of metabotropic GABAergic receptor signaling in the different phases of taste learning is yet to be comprehensively addressed.

Another mechanism involved in novel taste learning is NMDAR activation. Microinfusion of NMDAR antagonist APV into the IC inhibits correlative ERK activation following novel taste experience, indicating that converge on ERK activation following novel taste learning (Berman et al., 1998, 2000; Gutiérrez et al., 2003a). However, this is likely to represent divergent processes that operate under distinct time-scales during learning. Specifically, NMDAR activation is not necessary for novel taste learning and subsequent AN, since these learning paradigms were found sensitive to blockade of cortical mAChRs, but not NMDAR blockade (Gutiérrez et al., 2003a). Earlier studies using muscarinic agonist carbachol have demonstrated muscarinic neurotransmission to additionally modulate phosphorylation of the NR2B subunit of NMDAR, and to contribute to ERK activation during novel taste memory formation (Rosenblum et al., 1996). NR2B phosphorylation is necessary for taste learning, and has been shown to inversely correlate with taste novelty (Nunez-Jaramillo et al., 2008; Barki-Harrington et al., 2009). However, NMDAR antagonism does not impair incidental taste memory formation, suggesting that novel taste memory encoding itself is not dependent on NR2B-mediated ERK phosphorylation (Gutiérrez et al., 2003a).

Consistent with the above, more recent work has demonstrated convergence of NMDAR and dopamine signaling pathways onto ERK in the IC following taste learning (David et al., 2014; Ramirez-Lugo et al., 2015). Specifically, dopamine has been shown to bind to its D1 receptor and to interact with the Y1472 domain of the NR2B NMDAR subunit to activate ERK during novel taste learning (David et al., 2014). Furthermore, intraperitoneal administration of the D1R agonist SKF38393 in NR2B F1472 mutant mice (where tyrosine phosphorylation is not possible) was associated with a suppression of both NR2B and ERK phosphorylation in the IC (David et al., 2014), suggesting that phosphorylation of Y1472 of the NR2B subunit of NMDAR following dopaminergic D1 receptor stimulation is not only correlated with the increase in ERK activation but necessary for it.

Indeed, NR2B tyrosine phosphorylation after learning promotes intracellular redistribution of the NMDAR population that promotes taste memory encoding during acquisition (Barki-Harrington et al., 2009). Yet, transgenic NR2B F1472 mice only display increased neophobia and delayed AN that diminishes following the second taste exposure, whereas associative learning, as tested using the CTA paradigm, is normal (David et al., 2014). Thus, dopaminergic activation of ERK in the IC via the NR2B subunit appears to contribute to the encoding of information regarding novel stimuli that is required for the AN, but not for taste recognition upon reconsolidation or association of CS-US (Levitan et al., 2016). Glutamate and dopamine reactivation has been reported in the insula during the consolidation of CTA memories, 45 min following the administration of a malaise-inducing US (Guzman-Ramos et al., 2010; Guzman-Ramos and Bermudez-Rattoni, 2012). It is thus likely that NR2B-dependent ERK plasticity operates during memory consolidation to facilitate the encoding of information modulating the expression of behavioral adjustments to the novel tastant, rather than taste familiarity itself.

Increases in pERK are upstream of ELK-1, a member of the ternary complex factor present at the serum response element for the promoter region of several IEGs, including *c-fos* (Hipskind et al., 1991; Treisman, 1995). The induction of IEGs in the IC is regulated by the parallel activation of plasticity-related signal transduction pathways (Table 3). For instance, in the case of *c-fos*, activation increases in response to novelty and diminishes with familiarity, near mirroring effects observed on pERK (Koh and Bernstein, 2005; Yefet et al., 2006). During novel taste memory learning, ELK-1 transcriptional activity in the IC induces the expression of the postsynaptic membrane-95, membrane-associated guanylate kinase - PSD-95 (Yefet et al., 2006; Elkobi et al., 2008). PSD-95 is known to interact and regulate NMDAR trafficking and function (Lavezzari et al., 2003; Cousins et al., 2008). During novel taste learning, PSD-95 binds to phosphorylated NR2B and has been suggested to move along with the NMDAR into lipid raft membrane domains (Dong et al., 2004; Barki-Harrington et al., 2009). Increased pERK-dependent transcriptional activity in the IC is thus essential in promoting structural modification of the synapse, required for the acquisition and persistence of taste memories.

**Table 3:** Gene Expression at the IC modulated by Taste Learning.

**Learning paradigm**	**Gene**	**Effect**	**References**
Novel Taste Learning	Arc	Lateralization	Inberg et al., 2013
	Arc	↓	Morin et al., 2011
	Arc	–	Saddoris et al., 2009
	C/EBPβ	↑	Merhav et al., 2006
	c-fos	↑	Koh and Bernstein, 2005
	c-fos	↑	Yefet et al., 2006
	Homer	–	Saddoris et al., 2009
CTA Acquisition	Arc	↑	Barot et al., 2008
CTA Consolidation	Bdnf	↑	Ma et al., 2011

Current data on novel-taste memory acquisition would suggest ACh-dependent ERK activation in the IC to remove plasticity constraints (Table 2), and to shift proteostasis toward more protein synthesis while reducing synaptic protein degradation (Rosenblum et al., 1993, 2000; Rosenberg et al., 2016b). These two effects of mAChR signaling converge on p70S6K, and both shift proteostasis toward more synthesis during a relatively short time period of acquisition. Increases in protein synthesis driven by mAChR-dependent ERK activation in the IC are required for taste memory formation and are sufficient for AN (Levitan et al., 2016; Rosenberg et al., 2016b). Conversely, decreased protein synthesis facilitates memory retrieval that is resistant to IC NMDAR antagonism (Levitan et al., 2016). Whereas, the induction of mAChR-dependent protein synthesis through ERK, minutes following a sensory experience, is required for the acquisition of novel taste information by the IC, NMDAR – CaMKII dependent up-regulation of proteasome activity 4 h later is necessary for associative learning (Rosenberg et al., 2016a,b).

Interestingly, timely decreases in mAChR-dependent ERK proteostasis activation during retrieval could be equally instrumental to memory maintenance (Rappaport et al., 2015). For example, mRNA expression of the endogenous negative regulator of taste learning NRH: quinone reductase 2 (QR2) (Benoit et al., 2010) was found to be down-regulated in a mAChR-dependent, NMDAR independent manner, and to promote taste memory stabilization (Rappaport et al., 2015). The mechanism through which mAChR signaling reduces QR2 expression to allow stronger memory acquisition remains unknown, but has opened a number of possibilities relating to its potential as a target for improved memory and cognition.

## Aversive taste memory encoding and the insula

Studies of learned aversion in the laboratory typically involve association of known appetitive novel tastants, such as sweet or salty water, with gastric discomfort (Nachman, 1970). Intra-peritoneal administration of LiCl stimulates sensory portions of the vagus and splanchnic nerves innervating the gut producing signs of illness that serve as the US (Niijima and Yamamoto, 1994). Following CS-US pairing CS intake is suppressed, suggesting a conditioned downshift in taste palatability (Bures et al., 1998). Aversion to a number of bitter substances such as quinine and phenylthiocarbamide is unconditional in mammals, mediated through innate hereditary mechanisms (Kutscher and Wright, 1977; Kutscher et al., 1977; Whitney and Harder, 1986). The hereditary component of this behavioral response is understood to underlie sensory differences among individuals independent of the conditions associated with the initial encounter of stimulus in question. In this section we will focus on acquired taste responses and in particular CTA.

Conditioned and unconditioned responses to tastants differ in the neural processes that govern their expression (Dunn and Everitt, 1988; Spector, 1995; Spector and Glendinning, 2009). CTA acquisition, prior to permanent or pharmacological inactivation of the IC produces CTA amnesia and enhances latent inhibition of CTA in rats, even though familiarity can still be acquired (Moraga-Amaro et al., 2014). However, large areas of the IC can be damaged without affecting CTA memory (Hashimoto and Spector, 2014; Schier et al., 2014; Blonde et al., 2015). For example, specific bilateral lesions in the dIC and aIC were associated with significant CTA expression deficits, whereas lesions of the gIC were without effect, demonstrating regional specificity in the function of the IC during CTA memory formation (Schier et al., 2014, 2016; Lin et al., 2015). It could be surmised that reward, palatability and other safety signals generated in other regions during the encoding of appetitive taste memories are sufficient for the maintenance of appropriate behavioral responses for familiarity during retrieval. In a sense, the role of the IC may not be taste discrimination, but rather the shaping of complex behavioral responses through off-line computations that allow association with interoceptive information, facilitating retrieval.

The involvement of the cholinergic system in CTA acquisition was first reported more than 30 years ago, through the use of relatively large doses of the muscarinic antagonist atropine in rats (Kral, 1971; Deutsch, 1978). Cholinergic innervation arising from the basal forebrain complex is crucially involved in the ability of rats to learn aversively motivated tasks (Lopez-Garcia et al., 1993). Cholinergic and GABAergic innervation of the cortex arising from the basal forebrain complex was demonstrated to be necessary for the acquisition and consolidation of inhibitory avoidance memories (Lopez-Garcia et al., 1993; Miranda and Bermudez-Rattoni, 1999). This finding was extended to CTA acquisition, in studies where lesions of cholinergic basal-cortical and basal-amygdalar projections from the NBM to the IC disrupted CTA acquisition in rats (Gutiérrez et al., 1999b,c; Hasselmo and Giocomo, 2006).

Even though systemic scopolamine administration is of no consequence to CTA retrieval, its microinjection into the IC during the first 4 h following learning does abolish taste aversion for several days (Naor and Dudai, 1996; Berman et al., 2000; Gutiérrez et al., 2003a). Notably, an effect on short term memory and long term memory is only observed when microinjections are carried out prior to taste presentation during acquisition, but not prior to retrieval testing (Ferreira et al., 2002). Early and more recent studies have demonstrated mAChR- and dopaminergic NR2B-dependent signaling, to promote ERK activation minutes following novel taste consumption (Berman et al., 2000; David et al., 2014). Muscarinic activation of protein synthesis in the IC downstream of ERK following novel taste exposure is thus required in both appetitive and aversive memory acquisition (Nunez-Jaramillo et al., 2008). Pairing a stimulus with the release of either ACh (Gutiérrez et al., 1999b,c), or dopamine (DA) evokes long lasting increases in the responses of neurons to the paired stimulus (Bao et al., 2001; Schultz, 2010; Zellner and Ranaldi, 2010). Recent computational studies examining the representation and classification performance of a neural network, indicate ACh and DA to trigger distinct changes in neural representations to improve performance (Holca-Lamarre et al., 2017). Based on model data, the authors suggested that ACh redistributes stochastic neuronal selection so that more neurons encode stimulus classes that are challenging for the network, while DAergic signals adapt synaptic weights to match the classes of the task at hand.

Early pharmacological studies (Table 1), utilizing microinfusion of GABAA receptor antagonist bicuculline and GABA analog muscimol demonstrated GABAergic modulation of both CTA acquisition and retrieval (Berman et al., 2000). Both bicuculline and muscimol microinfusion into the IC prior to CTA training impaired subsequent acquisition and latent inhibition of CTA, but only muscimol elicited a deficit in CTA retrieval (Miranda et al., 2000; Moron et al., 2002). Inhibition of ERK phosphorylation in the IC by GABA likely hinders the interception of CS-related cues that encode salience and facilitate cognition required for CTA retrieval (Berman et al., 2000). Transgenic mice lacking specific subtypes of the postsynaptic metabotropic, revealed that GABAB(1a) knockout mice fail to acquire CTA, while GABAB(1b) knockouts fail to extinguish CTA (Jacobson et al., 2006). Systemic administration of the GABA_B_ agonist baclofen has been demonstrated to act as an US for CTA training (Wilson et al., 2011), even though others have argued otherwise (Chester and Cunningham, 1999). Whether the aforementioned GABAB-dependent effects relate to activity within the insula is still under investigation.

GABAergic neurotransmission modulates activity in the IC through local networks, in concert with inputs from the NBM (Wood and Richard, 1982; Zaborsky et al., 1986) or through GABA_A_ receptor activation in the NBM alone (Chu et al., 1990; DeSousa et al., 1994). Others have argued that the acquisition of CTA is primarily dependent on the parabranchial nucleus, which is the region in control of the release of familiarity and safety encoding GABA at the IC (Reilly, 1999). In studies where a number of brain regions thought to be involved in CTA acquisition and retention, dramatic deficits in the behavioral paradigms were induced by lesions of the PBN, the thalamus and the BLA, as well as the GC and BLA in this order (Yamamoto et al., 1995). Our view is that the role of the GC cannot be limited to novelty detection or discrimination, as others have supported, but we also propose the GC to act as a hub, and not a “stand-alone” structure in the formation and persistence of internal representation relating to taste aversion.

Evidence of the rule of glutamate in CTA acquisition was first demonstrated by the systemic administration of non-competitive and competitive NMDAR antagonists prior to saccharin exposure (Welzl et al., 1990; Willner et al., 1992). Glutamatergic projections to the IC from the amygdala are currently well recognized for their role in aversive taste memory formation (Everitt and Robbins, 1997; Gutiérrez et al., 2003b; Ferreira et al., 2005). Injection of LiCl or the arrival of appropriate malaise-reporting visceral inputs in the subsequent hour following taste exposure elicits increases in glutamate release in the IC that is instrumental to CTA memory consolidation (Table 1). *In vivo* microdialysis and capillary electrophoresis studies have suggested these effects in the IC to be downstream of amygdala-dependent glutamate and dopamine reactivation 45 min following CTA learning (Guzman-Ramos et al., 2010; Guzman-Ramos and Bermudez-Rattoni, 2012).

BLA-IC LTP enhances CTA memory persistence, but can be blocked by intraperitoneal administration of NMDAR antagonists 3 (2-carboxypiperazin-4-yl) propyl-1 phosphoric acid (CPP) and dizocilpine maleate (MK-801) (Escobar and Alcocer, 1998; Escobar and Bermudez-Rattoni, 2000). NMDAR blockade in the IC prior to taste presentation is only of consequence to CTA long term memory but not short term memory, consistent with the temporal time frame of NMDAR-dependent LTP (Ferreira et al., 2002). The characteristic role of the NMDAR during LTP is thought to underlie the encoding of associative signals essential for CTA consolidation following acquisition training (Davis et al., 1992; Escobar et al., 1997). Bilateral microinjection of the NMDAR antagonist 2-amino-5-phosphonopentanoic acid (AP5) into the IC, however, disrupts CTA memory formation, but is inconsequential to incidental taste learning (Gutiérrez et al., 1999a, 2003a). In further support, NMDAR antagonism of the IC was shown to disrupt both short term memory and long term memory of CTA only when carried out following LiCl administration (Ferreira et al., 2005). As more recent studies in rats demonstrated, NMDAR-dependent signaling is required for the maintenance of a short-term (3 h) memory trace in the IC, that is required for the development of the association between the CS and US (Adaikkan and Rosenblum, 2015). This NMDAR-dependent IC memory trace modulates CTA acquisition within conditioning inter-stimulus intervals (ISI) of up to 3 h (Adaikkan and Rosenblum, 2015).

Tyrosine phosphorylation of the NMDAR subunit NR2B and NMDAR-dependent increases in PSD-95 have also been reported to increase in response to CTA training (Rosenblum et al., 1995; Miyakawa et al., 1997). NR2B phosphorylation in response to CTA training was shown to be a function of the novelty of the taste stimulus, the quantity of the taste substance consumed, as well as the efficacy of the taste as a CS to become aversive (Rosenblum et al., 1995; Miyakawa et al., 1997).

LTP in the BLA-IC projection prior to CTA training facilitates its retention, while promoting a protein-synthesis dependent suppression of LTP in the BLA-IC pathway in the subsequent 120 h (Escobar and Bermudez-Rattoni, 2000; Rodriguez-Duran et al., 2011). This characteristic effect of CTA acquisition on subsequent IC plasticity is susceptible to local blockade of the NMDAR, as well as the inhibition of PKC (but not PKA) (Rodriguez-Duran and Escobar, 2014). NR2B tyrosine phosphorylation during acquisition is thus likely to prime activated synapses to incorporate circuit-wide associative input during consolidation through NMDAR-dependent synaptic potentiation (Ferreira et al., 2005).

Importantly, the development of this short-term memory trace during novel taste learning alone correlates with up-regulation of T286 CaMKIIα phosphorylation in synaptosomal fractions of the IC (Adaikkan and Rosenblum, 2015). However, NMDAR-dependent T286 CaMKIIα phosphorylation is dispensable to incidental taste learning itself, and only influences the maintenance of the CS-US association during CTA training with ISIs of up to 3 h (Adaikkan and Rosenblum, 2015). A separate cluster of rats acquired significant, but weaker aversion to saccharin even during conditioning with an ISI of 4–8 h, which was not susceptible to inhibition of IC CaMKIIα. Behavioral responses observed in this cluster of animals exhibit similarities to animals that were subject to pharmacological inhibition of components of the plasticity-related signaling in the IC (Rosenblum et al., 1993; Barki-Harrington et al., 2009; Parkes et al., 2014). In addition, increased susceptibility to extinction was observed in the ISI of 5–8 h compared to the ISI of 1–3 h clusters (Adaikkan and Rosenblum, 2015). This and other findings (Table 2), thus suggest the balance of NMDAR-dependent and independent plasticity at the IC to influence the dominance of aversive memory traces (Koh et al., 2009; Hashikawa et al., 2013; Marotta et al., 2014).

Other components of the glutamatergic system, such as ionotropic AMPA receptors incorporate plasticity-related signals from lower brain centers (Berman et al., 2000; Yasoshima et al., 2000). For example, glutamatergic release from the BLA to the IC during CTA retrieval is attenuated by both NMDAR and AMPAR-antagonism (Yasoshima et al., 2000). However, CTA retrieval is only susceptible to AMPAR antagonism of the IC, unlike other forms of glutamatergic receptor antagonism (Berman et al., 2000). Furthermore, unlike blockade of the NMDAR-CaMKIIα effects, CNQX (competitive AMPA/kainate receptor antagonist) microinfusion into the IC 1 h following novel taste learning suppresses CTA retrieval even when the US is delivered 4 h later (Adaikkan and Rosenblum, 2015). On the other hand, microinfusion of CNQX into the amygdala disrupts CTA expression and suppresses norepinephrine and dopamine augmentations in the IC (Osorio-Gomez et al., 2016). Novel taste learning itself induces up-regulation of the GluR1 AMPAR subunit, while AMPAR trafficking and signaling in the IC influences the ability of long-term memory trace inputs to form negative CS-US associations (Adaikkan and Rosenblum, 2015; Osorio-Gomez et al., 2016). AMPAR-dependent activity in the IC might underlie the ability of the brain to encode the aversion expected by a sensory experience and to re-evaluate its relevant weight in predicting the US in relation to inputs arising from the extended taste learning circuit upon subsequent consolidation trials (Eisenberg et al., 2003).

CTA acquisition, but not retrieval, can also be disrupted by either mGluR5-specific or Group II-III mGluR antagonist (MCPG) IC infusion prior to training (Berman et al., 2000; Bills et al., 2005). Conversely, only microinfusion of MCPG was associated with increased aversion during latent inhibition testing (Berman et al., 2000). More recently, microinfusion of mGluR1 antagonist AIDA into the IC at 0, 30, but not 60 min prior to taste presentation accelerated the AN in rats, whereas analogous infusion of glutamate impaired AN (Ramirez-Lugo et al., 2015). GluR1 activation following taste learning likely relates to the expression of a time-restricted warning signal in the IC, which restrains\s uncontrolled intake upon encounter of a novel stimulus in fear of potentially unsafe post-ingestive consequences (Ramirez-Lugo et al., 2015).

Findings might be indicative of opposing roles for Group I and Group II-III mGluRs during taste memory formation. Group I mGluR activation in the IC appears to interact with CS-dependent and preceding US-dependent glutamatergic neurotransmission to promote CTA learning through BLA-IC LTP (Guzman-Ramos and Bermudez-Rattoni, 2012). Given that latent inhibition is inhibited by MCPG and the opposing effects of Group I and Group II-III mGluR signaling on cAMP (Barros et al., 2000; Berman et al., 2000; Page et al., 2006), it is possible that regulation of cAMP through mGluRs at the IC modulates the formation of associations during acquisition (Escobar et al., 2002). However, distinct processes might dictate AN and LI, and the role of Group II-III mGluRs in appetite taste memories should be examined separately.

Interestingly, recent studies of LTP and LTD in slices of the BLA have demonstrated that application of mGluR1 agonist DHPG promotes the induction of LTP following single pulse theta-burst stimulation (Chen et al., 2017). Palatability signals generated in the BLA and relayed to the IC could thus incorporate this early novelty-detecting warning signal through mGluR-dependent mechanisms (Chen et al., 2013; Ramirez-Lugo et al., 2015). The reported priming effect of DHPG on subsequent BLA LTP is susceptible to mGluR5 antagonism, is PLC-dependent, and partially dependent on PKC (Chen et al., 2017; Rodriguez-Duran et al., 2017). The suppression of IC LTP following CTA acquisition is susceptible to local blockade of the NMDAR, as well as the inhibition of PKC (but not PKA), that could further implicate mGluR signaling (Rodriguez-Duran and Escobar, 2014). mGluR-dependent regulation of PKC levels in the IC during novelty might participate in CTA memory consolidation through BLA-IC LTP (Rodriguez-Duran and Escobar, 2014; Ramirez-Lugo et al., 2015).

## Learning and re-learning in the insula

Memory consolidation, a progressive process of post-acquisition stabilization that facilitates the permanent storage of memory, is dependent on the gene expression and synthesis of new proteins within relevant brain structures (Davis and Squire, 1984; Kandel, 2001, 2012). A diverse repertoire of plasticity is expressed in glutamatergic synapses in response to synaptic input, including several mechanistically distinct kinds of synaptic potentiation and depression, as well as homeostatic synaptic scaling (Lee et al., 2005; Park et al., 2006; Wibrand et al., 2006). Activity-dependent local synthesis and degradation of proteins is thought to translate synaptic inputs into remodeling of synaptic composition, and dendritic spine function (Steward and Shuman, 2003; Bramham and Wells, 2007).

Plasticity-related molecular signaling regulates transcription factors such as CREB and Elk-1 (Table 3), regulate IEGs shaping the genetic program through which memories are consolidated (Guzowski et al., 1999; Okuno, 2011). Induced by CREB-dependent transcription, the brain-derived neurotrophic factor (BDNF) regulates neuronal activity, differentiation, and learning-related plasticity (Barde et al., 1987; Leibrock et al., 1989; Huang and Reichardt, 2001; Chao, 2003). BDNF modulates synaptic plasticity through phosphorylation of the NR1 and NR2B NMDAR subunits via PKC and ERK (Iwasaki et al., 1998; Mizuno et al., 2003). Importantly, BDNF promotes AMPAR trafficking and stability at the synapse, via PKC and CAMKIIα (Wu et al., 2004), inhibiting GABAergic inhibitory post-synaptic potentials (Henneberger et al., 2002).

Inhibition of BDNF protein secretion or signaling impairs memory acquisition, retention, and recall in hippocampus- and amygdala-dependent learning paradigms (Linnarsson et al., 1997; Minichiello et al., 1999; Alonso et al., 2002; Rattiner et al., 2004). Studies of the role of BDNF signaling in CTA acquisition, demonstrated CREB-dependent increases in the CeA and IC to occur 12 h following conditioning (Swank, 2000; Desmedt et al., 2003; Ma et al., 2011). BDNF infusion into the IC, previously known to revert CTA memory impairments induced by protein inhibition (Miguel-Gonzalez et al., 2008), was more recently shown to convert a weak CTA to a strong one when administered following training (Martinez-Moreno et al., 2016). The induction of BDNF transcription is thus likely to facilitate aversive taste memory formation through BLA-IC LTP, while BDNF protein expression in the IC appears to confer memory maintenance and stability of predicted CS-US associations.

Another important target of CREB transcription is the gene coding for the activity regulated cytoskeleton-associated protein, *Arc/Agr3.1* (Guzowski et al., 2000; Waltereit et al., 2001). *Arc* is an activity-regulator effector IEG that exhibits dependence on excitatory glutamatergic transmission (Link et al., 1995; Lyford et al., 1995; Guzowski et al., 2001). Extensive studies in the rat hippocampus have indicated *Arc* induction and translation to be required for AMPAR endocytosis typically observed during the transition from LTP to LTD (Guzowski et al., 2000; Chowdhury et al., 2006; Plath et al., 2006; Bramham et al., 2008). Interactions of Arc with endocytic proteins endophilin and dynamin, form the post-synaptic trafficking endosome for AMPARs, essential for homeostatic plasticity maintenance (Chowdhury et al., 2006). In activated excitatory synapses, mGluR-dependent LTD induction is facilitated by increases in elongation factor 2-dependent translation of newly activated *Arc*, promoting cytoskeletal adaptations required for LTP consolidation (Kanhema et al., 2006; Messaoudi et al., 2007; Park et al., 2008; Jakkamstti et al., 2013).

*Homer1a*, much like *Arc*, is enriched in excitatory synapses and its translation mediates structural changes at the post-synaptic density that are instrumental to activity-dependent neuronal plasticity and development (Brakeman et al., 1997; Xiao et al., 1998; Garner et al., 2000). Constitutively expressed *Homer* isoforms bind to mGluR1 and link to the postsynaptic density through C-terminal coiled-coil (CC) multimers (Brakeman et al., 1997). The IEG Homer1a, however, is an inducible truncated isoform that inhibits CC multimer formation. This in turn promotes mGluR interactions with endoplasmic-reticulum resident inositol triphosphate receptors (IP3Rs), leading to enhanced Ca^2+^-dependent intracellular activity (Tu et al., 1998, 1999). In addition, *Homer1a* complexes with PSD-95 and Shank, to physically link mGluR5 and NMDAR-signaling, an important event during late phase LTP (Naisbitt et al., 1999; Fowler et al., 2011).

*Arc* and *Homer1a* are NMDAR-dependent plasticity markers that exhibit differential temporal expression patterns in activated neurons (Guzowski and Worley, 2001; Guzowski et al., 2001). In the BLA itself, CS- and US-specific increases in *Arc* mRNA have been demonstrated minutes following CTA training (Barot et al., 2008). Similarly, in the IC, *Arc* as well as *Homer1a* mRNA expression is differentially up-regulated within minutes of incidental novel but not familiar sucrose tasting (Saddoris et al., 2009). Furthermore, indicative of a role in the formation of predictions during retrieval through IC IEG induction, presentation of CS-related odor cues alone induced similar increases in *Arc* and *Homer1a* to the CS itself (Saddoris et al., 2009). Importantly, lesions of the BLA only attenuated, but did not inhibit this effect, suggesting the induction of NMDAR-dependent IEGs at the IC encompass information out with palatability and hedonic value inputs arising from the amygdala.

Okuno et al. have demonstrated Arc to be captured at less active synapses, a phenomenon known as inverse synaptic tagging (Okuno et al., 2012). This localization of Arc is mediated by high-affinity binding to inactive CaMKIIβ, resulting in AMPAR endocytosis and spine shrinkage (Kim et al., 2012; Okuno et al., 2012). Studies indicate that such signaling events are likely to be key to the formation of the short-term taste memory trace at the IC, that is required for CS-US association and effective CTA memory formation (Adaikkan and Rosenblum, 2015). Even though elegant studies using catFISH were not able to demonstrate coincidence detection of CS and US through changes in subcellular *Arc* mRNA expression at the IC, it is likely that occur at different times points at the BLA and the IC toward the maintenance of homeostatic scaling (Turrigiano, 2012; Vitureira et al., 2012). It would particularly interesting to examine how manipulation of signaling events such as NMDAR-CAMKII-AMPAR (Adaikkan and Rosenblum, 2015) or MAPK pathway (Elkobi et al., 2008), affect the memory process through changes in *Arc* transcription, translation and sub-cellular localization (Bluthgen et al., 2017; Nikolaienko et al., 2017a,b).

Conversely, studies have argued for CREB-independent gene transcription in the IC during novel taste learning (Swank, 2000; Desmedt et al., 2003; Yefet et al., 2006). Despite induction of cytoplasmic *Arc* mRNA at the IC following CS administration (Barot et al., 2008), consumption of novel sweet or salty water in rats had no overall effect on Arc protein IC expression 1 h following tasting (Inberg et al., 2013). Interestingly, Arc protein expression was lateralized 1 h following novel, but not familiar taste consumption. Increased Arc expression was observed in the right hemisphere in about 50% of the animals, whereas the remaining animals showed increased expression in the left hemisphere (Inberg et al., 2013). Importantly, Arc protein lateralization was evident in the background of significant ERK activation known to indirectly promote Arc transcription via CREB (Teber et al., 2004; Huang et al., 2007; Adaikkan and Rosenblum, 2012).

Complementary studies accounting for hydration-induced rapid increases in *Arc* mRNA in the IC, demonstrated novel taste learning to promote prolonged suppression of *Arc* transcription (Inberg et al., 2016). Rapid induction of pERK upon stimulation and the sharp initial induction of *Arc* mRNA in response to hydration alone might suggest ERK activation to underlie drinking-induced increases in *Arc* mRNA independently of taste learning-induced suppression (Inberg et al., 2016). Novel taste learning-induced suppression of *Arc* mRNA extends up to 6 h following consumption, despite increases in the number of differentially modulated genes compared to water consumption (Inberg et al., 2016). Arc has thus served to highlight the requirement, but also the complexity and spatiotemporal precision of taste learning-related computations mediated by transcription in the IC (Tzingounis and Nicoll, 2006; Bramham et al., 2008).

Novel taste-induced decreases in mRNA expression could relate to necessary adjustments toward levels that are required for optimal synaptic plasticity, achieved upon acquisition of familiarity (Inberg et al., 2016). Given previous reports regarding the ability of BLA-IC LTP in CTA acquisition, *Arc* transcription in the IC might be specific to computations regarding the expectation of negative experiences (Jones et al., 1999; Ying et al., 2002; Kanhema et al., 2006). CTA memory formation and retrieval require CREB-dependent increases in *Arc* expression in specific neurons of the IC and BLA (Barot et al., 2008; Sano et al., 2014). CTA memory formation exhibits ionotropic NMDAR dependency, which is likely promotes CREB transcription via PKC (Miranda and McGaugh, 2004; Adaikkan and Rosenblum, 2012; Rodriguez-Duran and Escobar, 2014). Increased cortical Arc mRNA expression was reported in mice classified as slow learners, exceeding levels recorded by autoradiography in over-trained animals (Kelly and Deadwyler, 2002, 2003). Subsequently translated Arc protein in specific neurons of the IC, consistent with the inverse synaptic postulate regarding the consolidation and maintenance of aversive memories (Kim et al., 2012), decreasing the capacity for behavioral plasticity to the CS.

Indicative of a requirement for lateralization of IC Arc expression during incidental taste learning in rats, the effect subsides upon repeated exposures to the same tastant (Inberg et al., 2013). Furthermore, unilateral and bilateral infusions of the IC with protein inhibitors, induced similar impairments on CTA acquisition (Inberg et al., 2013). Lateralization of sensory responses, has also been reported in a number of species of the evolutionary tree (Galati et al., 2001; Royet and Plailly, 2004; Taha et al., 2007; Concha et al., 2012). In separate studies, lateralization of Arc protein expression in the BLA has been reported during the processing of positive and negative emotional learning conditions (Young and Williams, 2013). In this study, fear conditioning was associated with increased Arc expression in the right, but not in the left BLA, while reward magnitude increased Arc expression in the left, but not the right BLA (Young and Williams, 2013). Earlier studies of brain activity during taste exposure in humans, reported increased fMRI activity in the left IC of right handed individuals and vice versa (Faurion et al., 1999). More recent imaging studies in adult males, suggest lateralized computations to encode taste presence, pleasantness and concentration (Dalenberg et al., 2015).

The specific evolutionary benefit in such lateralized computations is still a matter of debate (Halpern et al., 2005; Lust et al., 2011). It is possible that Arc lateralization in the IC relates not only in the encoding of novelty of sensory stimuli during incidental taste learning, but might also represent a state permissive to re-learning, where the association of the CS-US association is still labile. Subsets of valence-specific engram-bearing neurons have been identified through their transcriptional profile in the amygdalar complex (Kim et al., 2016, 2017). The bidirectional connectivity of the IC with these valence-specific neuronal populations of the BLA and CeA is yet to be examined, and could be particularly revealing of circuit-wide adaptations in everyday life and disease-related transcriptional activity.

Other studies have reported increases in Arc protein expression in dendrites of the GC when comparing familiar and novel saccharin (Morin et al., 2011). This finding would not necessarily be inconsistent with aforementioned studies, reporting increases in Arc mRNA in response to incidental but not novel taste learning (Saddoris et al., 2009; Inberg et al., 2013, 2016), but might suggest differential modulation of familiarity and novelty through transcription and translation (Adaikkan and Rosenblum, 2015). When considering the role of Arc at the post-synaptic density (Park et al., 2008) and aforementioned characteristics of BLA-IC LTP during CTA (Ramirez-Lugo et al., 2015), Arc expression in the IC could be indicative of “what the subject knows about the experience” and to act as a restrain to further learning through favoring AMPAR endocytosis and mGluR-dependent LTD (Gil et al., 1997; Kimura, 2000).

Studies examining transcription and relevant protein expression during extinction and reinstatement of CTA might be more revealing of circuit-wide adaptations that underlie subjective interpretation of sensory experiences and more closely model natural settings. Extinction and reinstatement of conditioned responses is a dynamic process in which the activity of particular brain regions changes, depending on the extent to which the conditioned response has been extinguished (Bouton, 1994; Lee et al., 2013). Extinction and reinstatement of CTA have only been examined by a few studies, while the precise role of the insula in this presumably cortical-dependent paradigm remains elusive. Similarly to fear conditioning, plasticity and activity within the mPFC has been heavily implicated in CTAE (Morgan and LeDoux, 1993). Sakata et al. reported that mutant mice with selective disruption of activity-dependent, plasticity-promoting BDNF expression in prefrontal cortex, impairs the extinction of CTA (Sakata et al., 2013). Nonetheless some differences in temporal correlation of the engagement of relevant sub-regions have already been noted. For example, unlike fear extinction, CTAE depends on functional protein synthesis but not on NMDA receptor activation in the ventromedial prefrontal cortex (Akirav et al., 2006). In further support, Mickley et al. noted that unlike fear extinction (where Infralimbic mPFC increases in relation to Prelimbic), CTAE induces increased expression of c-fos in both the Infralimbic and Prelimbic mPFC (Mickley et al., 2005). In follow-up studies from the same group, unlike fear extinction and reinstatement, activation of the CeA, reflected by c-fos immunoreactivity was not affected by CTAE or increased by spontaneous recovery (Mickley et al., 2007). Furthermore, CTAE was correlated with increased c-fos expression in the GC and mPFC, which was suppressed by spontaneous recovery of CTA (Mickley et al., 2007).

CTAE represents an inhibitory form of learning modulated by GABAergic signaling at the IC (Akirav et al., 2006). Conversely, activation of PI3-K signaling was reported to contribute to CTA maintenance and reinstatement (Slouzkey et al., 2013). Interestingly, the microinfusion of the IC with BDNF following CTA acquisition, accelerated CTAE (Rodriguez-Serrano et al., 2014), which others have suggested to be at least in part BLA-dependent (Xin et al., 2014). BDNF in the IC has been suggested to initiate CTAE without altering the original memory-trace (Xin et al., 2014), and to facilitate plasticity encoding the hedonic value of the CS in the absence of reinforcement (Furini et al., 2014). When considering findings suggesting LTP and LTD BLA-IC projection to exert bidirectional modulation of behavior (Rodriguez-Duran et al., 2017), it would be particularly interesting to examine whether and how this circuit encompasses hedonic and emotional processing. Other studies have suggested activity within the mPFC-IC (Jezzini et al., 2013; Gonzalez et al., 2015; Baldo et al., 2016) and orbitofrontal cortex-IC (Ramirez-Lugo et al., 2016) circuits to influence how motivation and choice-related computations are shaped. The precise temporal and cell-type specific plasticity within the IC could thus be pivotal to circuit-wide dynamics, determining the hedonic value and dominance of subjective engrams modulating behavior (Vincis and Fontanini, 2016; Avery et al., 2017).

Understanding the role of plasticity synaptic within distinct regions and the extended taste circuit during aversive taste CTAE, hold particular potential in explaining how associations regarding the salience and hedonic value of stimuli are modified in relation to experience. Data accumulated thus far is largely correlative, but encouragingly studies are increasingly able to address causality at the circuit level (Kim et al., 2016; Rajasethupathy et al., 2016; Roth, 2016). Imaging studies in humans, have heavily implicating the IC in self-referential processes shaping conscious awareness (Kurth, 2010; Critchley and Seth, 2012; Pais-Vieira et al., 2016). Further studies of the local activity and functional connectivity of the IC thus hold particular promise in relation to their link to autism (Odriozola et al., 2016), panic disorders (Banzett et al., 2000; Brannan et al., 2001), anorexia nervosa (Rolls, 1989, 2005; Shelley and Trimble, 2004), pain insensitivity (Singh et al., 2006; Potvin and Marchand, 2008), schizophrenia (Allen et al., 2004; Stephane et al., 2010), as well as alcohol and nicotine addiction (Fuehrlein et al., 2014; Abdolahi et al., 2017; Addicott et al., 2017).

## Author contributions

All authors listed have made a substantial, direct and intellectual contribution to the work, and approved it for publication.

### Conflict of interest statement

The authors declare that the research was conducted in the absence of any commercial or financial relationships that could be construed as a potential conflict of interest.
